# Cellular Models and Functional Assays for Assessing CFTR Function: A Comprehensive Review

**DOI:** 10.3390/ijms27125497

**Published:** 2026-06-18

**Authors:** Margarita Lopatina, Anna Demchenko, Svetlana Smirnikhina

**Affiliations:** Research Centre for Medical Genetics, Moskvorechye, 1, 115522 Moscow, Russia; demchenkoann@yandex.ru (A.D.); smirnikhinas@gmail.com (S.S.)

**Keywords:** cystic fibrosis, CFTR, cellular model, functional assay

## Abstract

Cystic fibrosis (CF) is a genetic disorder caused by dysfunction of the CFTR chloride ion channel. Progress in molecular understanding and therapy development relies on advanced cellular models and robust assays for evaluating CFTR function. This review traces the evolution of in vitro models, from primary and immortalized cell lines to patient-specific induced pluripotent stem cells (iPSCs) and complex three-dimensional systems. These advanced models, including air-liquid interface (ALI) cultures, organoids, and microfluidic organ-on-a-chip platforms, enable recapitulation of tissue architecture, cellular heterogeneity, and key pathological features such as impaired mucociliary clearance and chronic inflammation. A critical component of CF research is the accurate functional assessment of CFTR activity. We compare established high-resolution techniques (patch-clamp, Ussing chamber) with high-throughput screening assays, including fluorescence quenching of halide-sensitive YFP assay and organoid swelling tests. The article provides a framework for choosing the most appropriate CFTR functional assay tailored to specific research goals.

## 1. Introduction

Cystic fibrosis (CF) is an autosomal recessive disorder caused by pathogenic variants in the CFTR gene on chromosome 7. This gene encodes a channel protein that mediates the transport of chloride ions across the cell membrane [1]. CFTR channel dysfunction impairs ion balance and fluid secretion, leading to thick mucus accumulation in the respiratory and digestive tracts, persistent infections, and progressive organ injury [2]. As of 25 September 2024, the website of the international CFTR2 project (https://cftr2.org) has annotated 1085 nucleotide sequence variants in the CFTR gene that are known to cause CF. These variants are classified according to their impact on the CFTR protein, which includes the following categories: no protein synthesis (e.g., W1282X, 2143delT, G542X, 1677delTA [2]); impaired protein folding (e.g., F508del [3]); defective channel regulation (e.g., G551D [4]); reduced channel conductance (e.g., R334W [5] and R347P [6]); reduced synthesis of normal protein (e.g., 3849 + 10kbC > T [7]); and accelerated protein degradation (e.g., 120del23 [7]).

CF therapy involves symptomatic management (antibiotics, mucolytics [8]) and disease-modifying CFTR modulator therapy. CFTR modulators are oral medications of two main types: Correctors (elexacaftor/VX-445, lumacaftor/VX-809, tezacaftor/VX-661, vanzacaftor/VX-121) increase the amount of mature CFTR protein at the cell membrane; Potentiators (ivacaftor/VX-770, deutivacaftor/D-IVA) enhance channel function [9]. The chemical structures of these correctors and potentiators are comprehensively illustrated in a recent review by Lipani et al. [10]. The most effective treatment strategy is the combination of potentiators and correctors. Currently approved combinations include the triple therapy elexacaftor/tezacaftor/ivacaftor (Trikafta in the US; Kaftrio in the EU), the recently approved triple vanzacaftor/tezacaftor/deutivacaftor (Alyftrek), as well as the earlier dual combinations lumacaftor/ivacaftor (Orkambi) and tezacaftor/ivacaftor (Symdeko), and ivacaftor monotherapy (Kalydeco) for gating mutations. CFTR modulator therapy is lifelong and carries significant limitations, including the risk of hepatotoxicity, respiratory complications, affective disorders [11], and drug–drug interactions [12]. These drawbacks drive the ongoing search for radical and long-term curative treatments [10,13].

## 2. Cellular Models of Cystic Fibrosis

**Figure 1:**
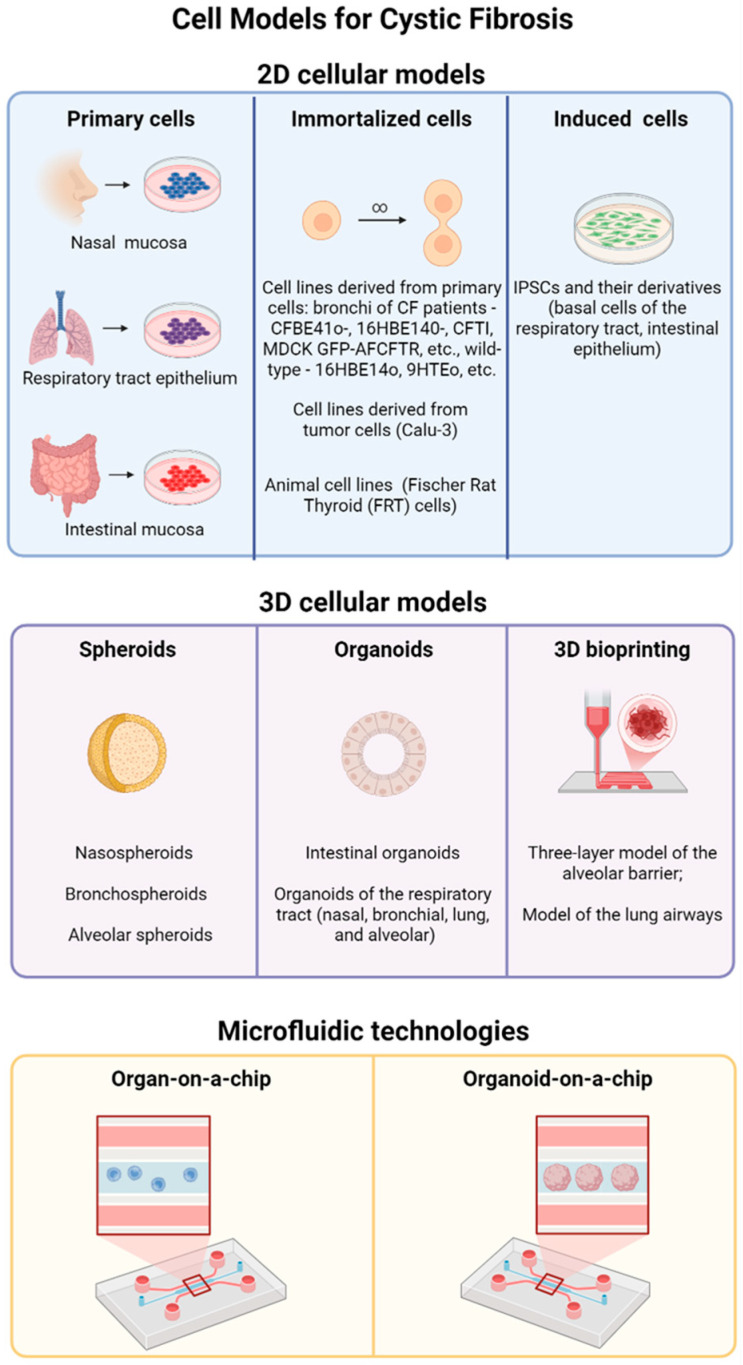
Cystic fibrosis cell models. Created in BioRender. Smirnikhina, S. (2026) https://BioRender.com/6418oor.

The development of CF cell models reflects the broader progress in biomedicine: a shift from simple two-dimensional (2D) cultures to complex three-dimensional (3D) systems that aim to recapitulate human tissue architecture and function in vitro [14,15]. This evolution is driven by several key challenges: studying molecular disease mechanisms, conducting high-throughput drug screening, and creating personalized platforms for therapy testing. The main CF cell models and their applications are summarized in Figure 1 and Table 1.

The initial stage involved primary cell cultures derived directly from patients (e.g., bronchial or nasal epithelium). These cultures retain the donor’s genetic and phenotypic profile, making them a “gold standard” for relevance. However, their use is limited by the invasiveness of the biopsy procedure, their finite lifespan in vitro, and significant scalability challenges [34,35,36].

Immortalized cell lines (e.g., CFBE41o-, Fischer rat thyroid (FRT) cells), capable of unlimited proliferation, solved the scalability issue and became a standard tool for molecular studies and primary compound screening [18,37]. However, the immortalization process and long-term culture lead to the accumulation of random mutations and the loss of tissue-specific characteristics, which compromises their physiological relevance [38,39]. Importantly, some cell lines commonly used in CF research do not express endogenous CFTR and must be engineered to do so. For example, Fischer rat thyroid (FRT) cells lack endogenous CFTR and are therefore a heterologous expression system requiring stable transfection with human CFTR cDNA. Similarly, certain airway-derived lines (e.g., CFBE41o-) have lost endogenous CFTR expression during immortalization and also require reconstitution.

A breakthrough came with the advent of induced pluripotent stem cells (iPSCs), which combine the advantages of previous models: they possess unlimited proliferative capacity while carrying a specific patient’s genome [40]. The differentiation of iPSCs into CF-relevant cell types (e.g., airway or intestinal epithelium) has paved the way for creating personalized in vitro models, studying disease pathogenesis, and developing gene-editing strategies (e.g., using CRISPR/Cas9) to correct mutations such as F508del [41,42,43,44]. It is also worth noting that undifferentiated iPSCs constitutively express the progesterone receptor at the protein level—a finding recently reported by Manganelli et al. [45]. Although progesterone is known to modulate CFTR expression and ion transport in certain epithelial tissues, this consideration pertains to the pluripotent stage prior to differentiation and does not directly affect CFTR functional assays performed in derived, differentiated cell types, which are the subject of this review.

Of particular value for evaluating therapeutic strategies, especially gene therapy, are chimeric (mixed) cultures. They model a realistic scenario in which corrected cells and cells with non-functional CFTR channels coexist within a tissue. Research using such models has established a crucial quantitative threshold: to restore approximately 40–70% of normal transport function, it is sufficient to have just 10–25% of cells expressing functional CFTR [46,47,48]. These findings provide critical target benchmarks for therapy development.

The most advanced stage of in vitro model development is represented by organoids (intestinal, nasal, bronchial) [13,43]. These self-organizing structures, grown from patient stem cells, recapitulate key aspects of tissue architecture, cellular heterogeneity, and polarity. Currently, the forskolin-induced swelling (FIS) assay in intestinal organoids has become a key functional test for personalized prediction of the response to CFTR modulators [49,50,51]. Respiratory organoids, derived from biopsies or iPSCs, represent an even more disease-relevant model for CF and are also beginning to be used for personalized therapy selection in cystic fibrosis [52,53,54]. In parallel, advanced bioengineering systems have emerged, including 3D bioprinting and microfluidic platforms (“organs-on-chips”). These technologies enable the integration of multiple cell types, the simulation of mechanical forces (fluid shear, airflow), and the study of complex pathological processes—such as impaired mucociliary clearance, biofilm formation, and chronic inflammation. These are hallmarks of CF pathology that cannot be adequately modeled in simple cultures [15,27,28,29,30,55].

3D bioprinting uses layer-by-layer deposition of cell-laden hydrogels. Ng et al. created a triple-layered alveolar barrier that was cultured at ALI to achieve differentiation and mucus production [29]. Kim et al. developed a 3D bioprinted tubular airway model in which mucociliary clearance was >3.5-fold slower in constructs derived from CF patients (homozygous F508del) than in healthy donor constructs, and was restored 2.5-fold upon treatment with Trikafta [28]. Practical adoption, however, faces several technical hurdles: bioink parameters require extensive optimization (cell density, temperature, pressure, curing time); although nozzle temperatures can briefly reach up to 300 °C, the microsecond exposure and rapid cooling prevent substantial damage to most human cell types under standard printing protocols—however, cell survival critically depends on cell type, bioink composition, and mechanical stress parameters (e.g., extrusion speed, pressure, nozzle diameter); creating intact epithelial barriers is challenging; batch-to-batch variability is high; and the approach is not suited for high-throughput screening [28,29].

Microfluidic technologies (including organ-on-chip and organoid-on-chip platforms) simulate airflow, mucus flow, and mechanical forces in nanoliter volumes, enabling study of complex CF pathologies such as impaired mucociliary clearance, biofilm formation, and chronic inflammation [56]. Plebani et al. developed a CF lung-on-a-chip that recapitulated mucus hypersecretion, P. aeruginosa colonization, and chronic inflammation [32]; Mazio et al. showed that lumacaftor reduces mucus viscosity using a reusable chip [30]; Mejías et al. adapted a vascularized model to 96-well format for high-throughput screening [31]. Organoid-on-chip (hybrid of organoids and microfluidics) offers dynamic microenvironment control while preserving 3D architecture; no CF-specific model exists yet, but platforms developed for other diseases (e.g., Hu et al. for lung cancer) could be adapted [33]. However, these technologies carry inherent drawbacks: fabrication is complex and expensive; PDMS chips absorb hydrophobic drugs (e.g., CFTR modulators); chip design varies widely, hindering standardization [57]; long-term culture and multi-organ integration remain difficult [30,31]. These technologies are not yet ready for routine application in clinical laboratory diagnostics of CF.

Thus, the modern research toolkit offers a diverse array of models, where the choice is dictated by the specific research objective. Immortalized cell lines are indispensable for high-throughput screening and mechanistic studies; personalized organoids and iPSC-derived models are ideal for therapy testing and investigating rare mutations; and engineered platforms are designed to model the systemic pathophysiology of the disease. However, developing an appropriate model is only the first step. A crucial final phase in any work aimed at correcting CFTR function is the precise and task-appropriate functional assessment of channel rescue.

## 3. Assays of CFTR Channel Function

The development and clinical implementation of CF therapies require reliable methods to assess the functional activity of the CFTR channel. Pathogenic variants in the CFTR gene lead to impaired chloride ion transport across the apical membrane of epithelial cells. Therefore, the quantitative measurement of this ion transport is essential for evaluating the efficacy of potential therapeutic compounds. Ion channels, including CFTR, mediate passive diffusion of ions driven by electrochemical gradients across the cell membrane; however, channel opening requires ATP binding and hydrolysis at the nucleotide-binding domains; measuring the resulting electrical current therefore enables direct, quantitative assessment of channel activity [58].

Modern methods for assessing CFTR channel function are based on two fundamental principles: the direct or indirect measurement of ion fluxes across cell membranes. Direct measurement involves real-time recording of ionic currents through a single channel or a population of channels in the cell membrane. Such methods quantify the electrical current carried by ions and provide the most precise information on channel kinetics, conductance, and regulation. Indirect measurement assesses channel activity by monitoring channel-induced secondary changes within the cell. Instead of measuring current, parameters such as transepithelial potential difference or changes in intracellular ion concentration (e.g., halides) are tracked. Indirect methods are often better suited for high-throughput screening—an automated approach for testing thousands of compounds for their ability to modulate target activity [59].

**Figure 2:**
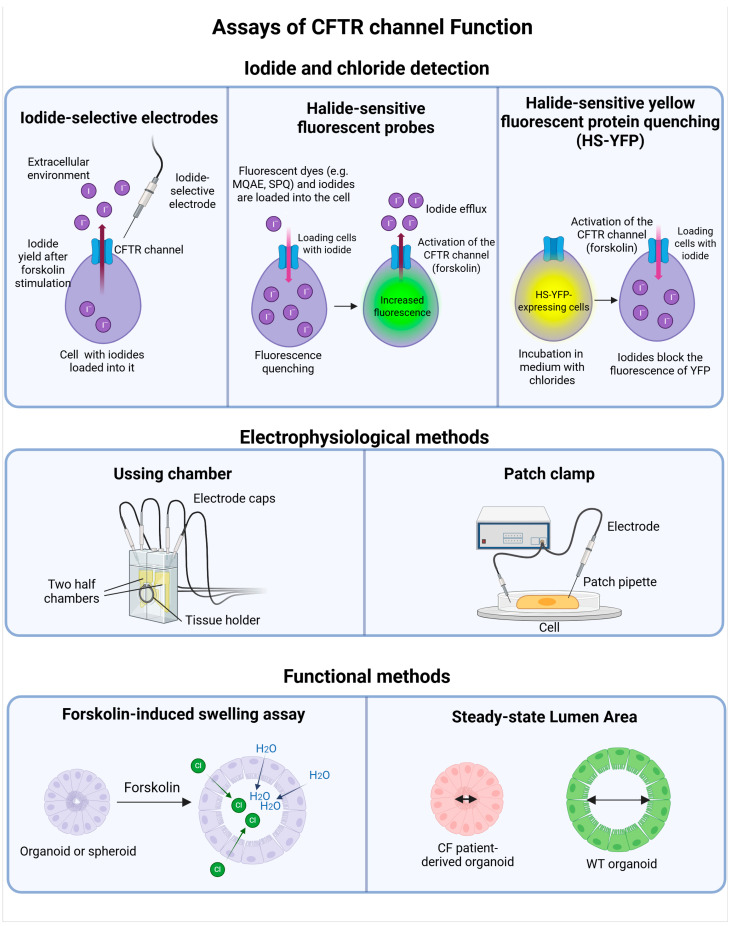
Methods for assessing CFTR channel function. Created in BioRender. Smirnikhina, S. (2026) https://BioRender.com/94kh647.

Approaches for evaluating CFTR channel function span from highly sensitive yet labor-intensive electrophysiological techniques (direct measurement), which record currents through single channels, to high-throughput screening assays (often based on indirect measurements), suitable for testing thousands of compounds. The choice of a specific method is governed by research objectives, required throughput, the type of cellular or tissue model, and the need for quantitative or qualitative data. Below we review the primary methods used to assess CFTR channel function in vitro, discussing their principles, advantages, limitations, and applications (Figure 2, Table 2).

### 3.1. Direct Measurement Methods

#### 3.1.1. Patch-Clamp

The patch-clamp technique enables the high-precision measurement of ionic currents across the cell membrane without causing significant damage. A glass micropipette (patch pipette) filled with an electrolyte solution is positioned near the cell membrane. Applying gentle negative pressure to the pipette forms a tight seal (gigaohm seal) with the membrane, providing mechanical stability and electrically isolating a small membrane patch for recording [5]. There are two primary approaches for performing measurements: manual and automated [69]. The classical manual method is the “gold standard” for fundamental research requiring the highest resolution, such as studying single-channel kinetics and biophysical properties. However, it is characterized by extremely low throughput, technical complexity, and requires a highly skilled experimenter. Automated patch-clamp systems were developed to address this speed limitation, as the processes of pipette positioning, seal formation, and signal recording are robotized. This adaptation makes the method suitable for high-throughput drug screening, functional characterization of numerous CFTR mutations, and validation of therapeutic compounds [70].

The patch-clamp technique also encompasses several configurations, with the choice dependent on the research objective. In the whole-cell configuration, after forming a tight seal, the membrane patch under the pipette is ruptured, providing direct access to the cell’s interior. This allows recording of the combined activity of all ion channels in the membrane. Because intracellular signaling cascades remain largely preserved, CFTR is typically activated by raising intracellular cAMP with forskolin (0.1–20 µM) and a phosphodiesterase inhibitor such as 3-isobutyl-1-methylxanthine (IBMX; 100 µM); in some protocols a membrane-permeable cAMP analogue (e.g., 8-CPT-cAMP) is used instead [71]. For instance, Noel S. et al. employed the whole-cell configuration to establish correlations between CFTR genotype, phenotype, and function in human nasal epithelial cells [60,72].

In the inside-out configuration, a membrane patch is excised from the cell, exposing the intracellular surface to the bath solution while the extracellular surface remains within the pipette solution. This method is particularly advantageous for studying CFTR as it enables the investigation of single-channel properties—such as the relationship between channel gating and protein conformational changes—and the effects of compounds like the potentiator GLPG1837 [60]. In this configuration, all soluble cytosolic components are lost; therefore, CFTR is directly activated by adding the catalytic subunit of protein kinase A (PKA, 20–100 nM) together with Mg-ATP (1–5 mM) to the intracellular (bath) solution. CFTR currents in inside-out patches are identified by their absolute dependence on PKA and ATP, and by their characteristic slow, voltage-independent gating that is potentiated by inorganic pyrophosphate [71].

The outside-out configuration involves excising a membrane patch so that the extracellular side is exposed to the bath solution, while the intracellular side is exposed to the pipette solution. It is used to study the influence of the external cellular environment on single channels. This configuration is rarely applied to CFTR research, as the vast majority of agents regulating the channel including all commonly used potentiators and correctors, act from the intracellular side [73].

Despite continuous refinement, both manual and automated patch-clamp approaches have inherent characteristics that must be considered. In the whole-cell configuration, the pipette solution dialyzes the cytoplasm. While this leads to the loss of some physiological soluble factors (which can be a limitation for studying processes that depend on native cytosolic components), it also provides a major advantage: the experimenter can precisely control the intracellular milieu (e.g., ATP, pH, calcium, nucleotides). This control is deliberately exploited to isolate CFTR-specific currents and to study channel regulation under defined conditions. Mechanical stress on the cell during seal formation and, most critically, the disconnection of the cell from its native tissue environment remains general limitations, as they preclude modeling of complex cell–cell interactions and long-term in vivo effects. Furthermore, the patch-clamp technique is highly operator-dependent, exhibits low inter-laboratory reproducibility, and lacks a standardized CFTR-specific protocol, which complicates cross-study comparisons and routine clinical implementation.

#### 3.1.2. Ussing Chamber

The Ussing chamber is a classical technique for the direct assessment of transepithelial ion transport. This method evaluates ion flux across an intact epithelial layer or a polarized cell monolayer grown on permeable supports. ALI-cultures are particularly physiologically relevant for such studies, as they faithfully recapitulate key properties of the in vivo airway epithelium. This allows for the study of ion transport under conditions that closely mimic the physiological state, as cells maintain their native architecture, polarity, and functional characteristics.

The method’s principle relies on measuring the short-circuit current required to maintain a zero transepithelial potential difference across the tissue sample separating the two halves of the chamber. To isolate the specific chloride current mediated by CFTR, the standard protocol involves blocking sodium transport via epithelial sodium channels (ENaC) on the apical side with the inhibitor amiloride [74]. Subsequent activation of CFTR with forskolin or similar agonists then allows for the quantitative assessment of the CFTR-specific contribution to overall ion transport.

Results from Ussing chamber studies often serve as a reliable predictor of CFTR modulator efficacy in clinical settings. This method successfully predicted the therapeutic potential of drugs like lumacaftor [75], ivacaftor, and tezacaftor [76]. Furthermore, the Ussing chamber is used to evaluate the success of CFTR gene editing, where functional correction is demonstrated by comparing short-circuit currents in edited samples to healthy donor controls. Restoration of chloride current in edited cells to levels statistically indistinguishable from non-CF controls provides direct proof of functional correction [77].

A key diagnostic application of the Ussing chamber principle is the intestinal current measurement (ICM) assay. This method measures the short-circuit current in a small rectal biopsy exposed to sequential pharmacological agents, each targeting a distinct ion transport pathway [61]. First, amiloride is applied to the apical surface to block ENaC, thereby eliminating the ENaC-dependent sodium current and electrically isolating the chloride transport component. Next, forskolin (a direct activator of adenylyl cyclase that raises intracellular cAMP) combined with IBMX (a phosphodiesterase inhibitor that prevents cAMP degradation) is applied to maximally activate CFTR via the PKA-dependent phosphorylation pathway; the resulting increase in short-circuit current directly reflects CFTR-mediated chloride secretion. Finally, carbachol (a cholinergic agonist acting on muscarinic receptors) is applied to stimulate calcium-activated chloride channels (CaCC), primarily TMEM16A/ANO1, providing an independent measure of non-CFTR chloride secretory capacity and serving as an internal positive control for tissue viability. This sequential pharmacological dissection allows the specific CFTR-dependent current to be unambiguously distinguished from other ion transport processes in the biopsy.

Despite its high physiological relevance, the Ussing chamber method has notable limitations. Firstly, its throughput is extremely low, allowing only a few samples to be processed per experiment, making it unsuitable for large-scale drug screening [78]. Secondly, it is a costly and technically complex system requiring specialized equipment and highly skilled personnel. Moreover, the method lacks a standardized CFTR-specific protocol. Considerable variation in CFTR-mediated currents has been reported across studies, owing to differences in culture conditions (e.g., passage number, ALI duration) and assay protocols (e.g., forskolin concentration, use of CFTR inhibitors) [79].

### 3.2. Indirect Measurement Methods

#### 3.2.1. Iodide-Selective Electrodes

The iodide-selective electrode method represents an indirect electrochemical approach for the quantitative assessment of CFTR channel function. Its principle relies on the ability of CFTR to conduct not only chloride ions but also other halides, specifically iodide (I−), which is normally absent in cells. After preloading cells with this anion, CFTR function is stimulated with forskolin. At specific time intervals (e.g., every minute), the extracellular medium is refreshed and collected to measure iodide efflux using an iodide-selective electrode [80]. The advantages of the method include high specificity for iodide detection and quantitative data output, enabling accurate estimation of CFTR-mediated anion transport rates and characterization of channel functional activity. This approach has been used to demonstrate the efficacy of various CFTR modulators [81]. This method is valuable for studying the fundamental properties of the channel; for instance, it helped establish that CFTR functions as an ion channel rather than an exchanger and defined its anion selectivity [82]. However, due to the larger ionic radius of iodide, its efflux from cells is slower and lower in magnitude compared to chloride, which can complicate the detection and quantification of anion transport via CFTR channels [62]. Additional limitations include low throughput and the technical complexity of the measurements.

#### 3.2.2. Halide-Sensitive Fluorescent Probes

This method measures changes in fluorescence associated with anion transport through the channel. Cells are loaded with a halide-sensitive fluorescent probe (e.g., MQAE [83] or SPQ [84]) and iodide [62]. Following iodide loading, fluorescence quenching occurs. Subsequently, the CFTR channel is activated (e.g., with forskolin), triggering iodide efflux from the cells via CFTR. The resulting decrease in intracellular iodide concentration leads to an increase in fluorescence, which is monitored using a fluorometer or fluorescence microscope. In this configuration, cells are pre-loaded with iodide, so the gradient favors efflux upon CFTR activation.

Importantly, this assay does not directly monitor chloride concentration. Instead, it uses iodide as a surrogate permeant anion to assess CFTR channel gating. This approach enables evaluation of channel function and the effects of various substances on CFTR activity by comparing fluorescence signals under different conditions. For example, using T84 cells expressing wild-type CFTR protein, researchers demonstrated the effect of a CFTR-stimulating cocktail containing dibutyryl cAMP, IBMX (3-isobutyl-1-methylxanthine), and isoprenaline [84]. The study described a method for assessing CFTR activity using a fluorescence spectrophotometer and the fluorescent probe SPQ as an indicator of anion transport.

#### 3.2.3. Halide-Sensitive Yellow Fluorescent Protein Quenching (HS-YFP)

This assay employs a genetically modified yellow fluorescent protein (YFP) with high sensitivity to quenching by iodide (HS-YFP) but significantly lower sensitivity to chloride. This is achieved through specific mutations, such as H148Q-I152L-F46L, in the YFP protein [63,85]. Approximately 5–10 min prior to measurement, forskolin is added to the cells to activate CFTR. Subsequently, an iodide-rich buffer is introduced. Because the extracellular iodide concentration is now high, the electrochemical gradient drives iodide into the cells. Iodide enters the cells through the activated CFTR channels, leading to a decrease in YFP fluorescence [85]. Compared to chemical halide indicators, YFP offers advantages such as non-invasive cellular loading and prolonged fluorescence [64]. This method is used to test the impact of various compounds on CFTR function. For example, Langron et al. demonstrated that the compound MS131A exhibits 50% of the activity of ivacaftor [86].

The detection of iodide and chloride ions is achieved through three main methodological approaches, each with distinct advantages and limitations. Methods utilizing iodide-selective electrodes offer direct and quantitative measurement of iodide efflux with high specificity but are characterized by low throughput and technical complexity. In contrast, fluorescence-based assays provide high sensitivity and are ideal for high-throughput screening in live cells. However, they are indirect, require careful calibration, and may suffer from phototoxicity and interference from cellular autofluorescence. In addition to methods for quantifying anion concentrations, equally crucial for CFTR research are functional assays that measure the physiological consequences of channel activity, specifically, the electrical current across the membrane or transepithelial ion transport.

#### 3.2.4. Forskolin-Induced Swelling (FIS)

The forskolin-induced swelling (FIS) assay is a standard functional test for assessing CFTR protein activity in intestinal [26] and airway spheroids [22,65]. Its principle is based on the activation of CFTR channels by forskolin, which drives the transport of chloride ions into the lumen of the structure. Water follows osmotically, causing the organoid or spheroid to swell—reflected as an increase in the area of the central lumen in organoids or in the overall diameter of the structure in spheroids, which typically lack a lumen; swelling here reflects global cellular expansion. A special case is nasal spheroids, which may form a small lumen [22]; in such “lumen-containing spheroids”, diameter increase is still measured). Thus, the change in cross-sectional area of an organoid or spheroid after forskolin addition serves as an indirect measure of CFTR channel functional activity.

A key practical advantage of 3D organoids over labor-intensive 2D transwell cultures (e.g., ALI cultures) is their high-throughput, which is critical for large-scale preclinical studies. While working with a single ALI culture typically allows only a single analysis, the 3D model enables large-scale parallel experiments. For instance, cellular material from just one ALI culture can be sufficient to generate hundreds of 3D organoids for subsequent testing in 96-well plates [87]. Objective and high-throughput analysis of such experiments requires appropriate equipment, such as live-cell imaging systems, which can automatically perform repeated imaging of organoids, along with software to calculate size changes. This allows for the quantitative evaluation of various interventions—including incubation with CFTR correctors and potentiators or the outcomes of gene therapy—based on the degree of restored swelling.

A major advantage of the FIS assay is its suitability for therapeutic screening. By incubating organoids with CFTR modulators or following gene therapy, the efficacy of pharmacological or genetic interventions can be quantitatively assessed based on the restoration of swelling. For example, the efficiency of CRISPR/Cas9-mediated correction of the pathogenic F508del variant in intestinal organoids was evaluated using FIS, showing a substantial 180–187% increase in total surface area in intestinal organoids [43].

To date, the intestinal organoid FIS assay has been firmly established as the “gold standard” for the personalized prediction of CFTR modulator therapy efficacy [26], including for patients with rare CFTR variants [66,88]. For respiratory tract organoids, the FIS assay enables the assessment of CFTR protein function and response to its modulators in a model closest to the airways. The analysis involves stimulating organoids with forskolin and incubating them with CFTR modulators (e.g., ivacaftor, lumacaftor) to determine whether channel function is restored [87]. The assay is performed by documenting changes in organoid size via microscopy and specialized software, calculating the relative increase in area. This approach allows for the evaluation of baseline CFTR function, prediction of therapeutic response, comparison of responses among different patients, and screening of new drugs [25].

However, the FIS protocol for respiratory organoids requires further optimization. This is because airway system organoids are characterized by slower growth and swelling kinetics compared to intestinal ones. Despite these technical challenges, the pulmonary model is considered more relevant for predicting therapy efficacy in the context of cystic fibrosis respiratory manifestations [51], making efforts to optimize it critically important.

#### 3.2.5. Steady-State Lumen Area (SLA)

The steady-state lumen area (SLA) measurement method is a functional assay for CF diagnosis based on assessing CFTR protein activity by measuring the steady-state lumen area within organoids. Unlike the FIS assay, this method does not involve forskolin stimulation. The procedure begins with generating organoids from patient-derived cells, such as intestinal biopsy stem cells, followed by cultivation under standard conditions. In the resulting structures, the lumen area is measured and expressed as a percentage of the organoid’s total area [89].

The primary diagnostic criterion is a significant difference in this metric between samples from healthy donors and CF patients: organoids from healthy donors exhibit a lumen area within a 35–70% range, whereas organoids with pathogenic CFTR gene variants typically show a lumen area of 0–10% [68]. This parameter reflects the ability of CFTR to regulate ion and water transport across epithelial cells, thereby assessing the degree of its functional impairment and aiding in distinguishing CF patients from healthy individuals. The test is used in preclinical research to evaluate the therapeutic effect of CFTR modulators.

However, compared to the more common FIS assay, the SLA method has lower sensitivity, limiting its widespread application [67]. A key limitation is its poor ability to differentiate the degree of CFTR dysfunction among patients with severe mutations, whose SLA values already fall within the low range (0–10%). Additionally, unlike FIS, which measures the relative size increase in response to forskolin stimulation, SLA assesses the baseline, ‘resting’ state. This makes SLA useful for comparing healthy and diseased organoids at rest but does not allow for a direct comparison of their response to stimulation [88,90,91]. However, these constraints do not preclude its utility in specific contexts. SLA can serve as a valuable complementary tool alongside FIS: while FIS captures the dynamic, stimulated response of CFTR, SLA reflects baseline channel activity under resting conditions, providing orthogonal information about residual CFTR function [90]. This makes the two assays particularly informative when used sequentially within the same organoid preparation. Moreover, SLA may be preferable in longitudinal studies where repeated forskolin stimulation is undesirable due to potential effects on organoid viability or signaling. Methodological refinements—including deep-learning-based automated image segmentation and standardized quantification pipelines—hold promise for improving the discriminatory power of organoid-based assays including SLA [88,92]. Thus, while SLA is currently less informative than FIS as a standalone assay for evaluating CFTR function and therapy efficacy, it remains a relevant tool within a multimodal assessment framework.

A critical practical consideration when selecting CFTR functional assays is their reproducibility and inter-laboratory variability. Electrophysiological methods (patch-clamp, Ussing chamber) are inherently operator-dependent: results are strongly influenced by cell culture conditions, passage number, and investigator experience, making cross-laboratory comparisons challenging.

The FIS assay has undergone the most rigorous multi-center validation: a four-laboratory European study demonstrated high intra- and inter-assay repeatability when a standardized SOP was followed, though longer-term reproducibility (>1 year) remained more variable, reflecting sensitivity to culture drift [93]. Key sources of variability in organoid assays include Matrigel batch, forskolin concentration, organoid passage, and image analysis pipelines—standardization of these parameters is essential for clinical application of FIS as a precision medicine biomarker [94].

Modern methods for assessing CFTR channel function comprise a diverse and complementary toolkit, enabling comprehensive investigation of cystic fibrosis pathogenesis and therapeutic efficacy. The reviewed techniques can be broadly divided into two categories: direct methods for measuring ion flux, such as patch-clamp and Ussing chamber, and indirect methods that evaluate the functional consequences of channel activity, including fluorescent assays, FIS, and SLA measurement. The choice of a specific method is guided by the research objective. Future development is focused on further miniaturization, automation, and the integration of different platforms. A particularly promising direction is combining microfluidic technologies with organoid models. This synergy will not only accelerate the development of targeted therapies for patients with diverse CFTR variants but also achieve a new level of physiological relevance while maintaining high screening throughput.

## 4. Selection of CFTR Assessment Methods Based on Research Objectives

Selecting the optimal method for assessing CFTR channel function is a pivotal step that determines the success of a research project. This choice involves finding the right balance between several key parameters: the project’s goal (from screening to personalized prediction), required throughput, analytical sensitivity and resolution, physiological relevance of the model, and practical constraints (biomaterial availability, equipment, time). An effective strategy often requires a combination of methods at different stages of the workflow [87]. Recommended approaches for various research objectives are outlined below. To facilitate a clear comparison of selection criteria, the methods are summarized in Table 3.

Fundamental research into channel biophysics requires methods that offer high resolution and detailed data. The most detailed insights are provided by the patch-clamp technique. In the inside-out configuration, it allows for the direct examination of the cytoplasmic side of the membrane and precise measurement of key biophysical parameters: channel conductance and current amplitude [72]. Iodide-selective electrodes also enable the assessment of the functional consequences of mutations or drug effects at the level of net cellular anion flux [80]. Their key advantage consists of generating absolute and reproducible kinetic curves of anion efflux, providing direct access to parameters such as transport rate and channel activation efficiency [82]. This method is particularly crucial for studying rare mutations when a quantitative correlation between the degree of functional defect and clinical phenotype is needed. Furthermore, it serves as a critical validation step following high-throughput screening, confirming a compound’s specific effect on CFTR-dependent transport through pharmacological modulation (e.g., evaluating the isolated contribution of a potentiator against a background of baseline activation). Despite requiring careful calibration, this approach remains a benchmark for the objective comparison of the functional integrity of various CFTR variants and the efficacy of therapeutic correctors and potentiators [81].

High-throughput screening of drug compounds critically relies on methods that combine automation, high-throughput, and a biologically relevant model [87].

To provide further historical context: the discovery of the first approved CFTR modulators (ivacaftor and lumacaftor) by Vertex Pharmaceuticals in partnership with the Cystic Fibrosis Foundation relied on two complementary high-throughput platforms. For potentiator discovery, a FLIPR-based fluorescence membrane potential assay on FRT cells stably expressing G551D-CFTR was employed [95]. For corrector discovery, a halide-sensitive YFP iodide influx assay in FRT cells expressing F508del-CFTR was used to screen approximately 150,000 compounds [96], with hit confirmation performed by Ussing chamber short-circuit current measurements. These campaigns established the HS-YFP and membrane potential assays as the industry standard for primary CFTR modulator screening.

The use of stable cell lines (e.g., CFBE41o-) ensures maximum standardization and speed for identifying top candidates. Another method is halide-sensitive fluorescent probes (e.g., SPQ and MQAE) that are also applied for similar screening tasks in 2D cultures. This method offers high sensitivity and is well-suited for the routine screening of modulators and the study of basic channel function in monolayer cultures [84]. When the task demands more detailed electrophysiological analysis while maintaining enhanced throughput, automated patch-clamp systems are employed. This method facilitates high-throughput characterization of CFTR mutations and validation of therapeutic compounds, serving as a bridge between large-scale screening and fundamental channel studies [70]. FIS assay in organoids is equally suitable for high-throughput screening. This method shifts screening into a biological context by using three-dimensional, patient-derived organoids [26]. It combines high-throughput (96-well plate format) with a genetically relevant, patient-specific model that preserves native tissue architecture and protein expression. This allows not only for evaluating compound efficacy but also for directly predicting their potential clinical benefit for specific genotypes—a direct step toward personalized medicine [15,66].

For validating the therapeutic efficacy of compounds and predicting clinical response, methods that provide physiologically relevant conditions and quantitative correlation with patient outcomes are paramount. At this stage, the Ussing chamber has established itself as an indispensable tool, its predictive power validated by the entire history of CFTR modulator development [75,76]. The measurement of short-circuit current (Isc) in this system provides a direct quantitative readout of net epithelial ion conductance [74]. This very parameter demonstrated a strong correlation with improved lung function in major clinical trials, solidifying the Ussing chamber’s status as the “gold standard” for final preclinical validation.

For personalized medicine and therapy selection in CF, methods capable of predicting an individual patient’s therapeutic response based on their own biomaterial become crucial, especially for carriers of rare CFTR mutations. The choice of tool for this diagnostic strategy is determined by the clinical objective and the availability of biomaterial. The standard for assessing the function of rare CFTR variants is the FIS assay on rectal organoids, which allows for therapy selection based on a correlation between the laboratory response and clinical outcome [26,55]. A less invasive alternative, particularly important for pediatric applications, is the FIS test on nasal organoids, for which material is obtained via brush biopsy, enabling early diagnosis and therapy monitoring [22,25,87]. The SLA measurement method is less preferred due to its lower sensitivity compared to FIS [68,88]. Alongside promising in vitro models, a classic ex vivo tool remains the intestinal current measurement (ICM). Its primary strength lies in its high sensitivity for resolving diagnostic uncertainty in cases with borderline sweat test results. Furthermore, ICM allows for direct, ex vivo assessment of a potentiator’s acute effect on patient tissue, providing functional proof of therapy response within hours [97].

Assessing gene therapy strategies for CF requires methods capable of detecting and quantifying restored CFTR function even when genetic intervention efficiency is low, while also functioning within physiologically relevant, tissue-specific models. This challenge is met by combining two key approaches, which together provide both high analytical sensitivity and biological validity. The central tool for precise quantitative assessment of correction is the Ussing chamber. This method becomes critically important in the context of gene therapy, where transduction or editing efficiency often does not exceed 1–15% [77]. Its unique advantage lies in its extremely high sensitivity, enabling the detection of CFTR-dependent current even with only 5–10% functionally corrected cells within a chimeric population. This is possible due to the signal amplification capability of a polarized epithelial monolayer: even a small number of cells expressing functional CFTR can coordinate ion transport across the entire epithelial sheet, which is recorded as a measurable short-circuit current. Thus, the Ussing chamber bridges a critical gap in gene therapy development, providing a means to prove concept and evaluate approach efficacy even at early, unoptimized stages. Also, for evaluating gene and cell therapy strategies, the FIS assay on organoids derived from edited iPSCs is employed. This platform assesses editing success not in isolated cells but within differentiated, tissue-specific structures—intestinal or, most relevantly, lung organoids [43]. This approach mimics key aspects of in vivo physiology: three-dimensional organization, cellular heterogeneity, and tissue-specific expression patterns. The FIS assay in this model serves as a relevant screening platform, allowing researchers to not only confirm correction but also to evaluate whether it leads to the expected functional outcome (swelling) within the appropriate tissue microenvironment. The synergy of these methods creates a complete evaluation cycle. The Ussing chamber offers quantitative sensitivity to prove the fundamental feasibility of a strategy even with low efficiency, while FIS analysis on iPSC-derived organoids provides biological validation of correction in a relevant tissue-specific system. This integrated approach enables a shift from simply confirming gene editing to demonstrating functional restoration at a level that predicts potential therapeutic benefit.

## 5. Conclusions

The choice of an optimal CFTR functional assay is not a one-size-fits-all decision but rather a strategic balance between experimental goals, required throughput, physiological relevance, and available resources. No single method simultaneously provides maximal resolution, high-throughput, and perfect tissue context. Instead, successful CF research increasingly relies on tiered workflows: high-throughput fluorescent or organoid-based assays for primary screening, followed by electrophysiological validation (Ussing chamber or patch-clamp) for mechanistic insight and preclinical confirmation, and patient-derived organoid assays for personalized therapying. As the field advances toward gene therapy and individualized medicine, integrating complementary assay platforms in physiologically relevant models will be essential for robust evaluation of CFTR-targeted approaches.

## Figures and Tables

**Table 1:** A comparison of cellular models for cystic fibrosis.

	Examples	Derivation	Advantages	Disadvantages	Applications	References
Primary cell cultures	Airway epithelial cells; Intestinal cells; Nasal mucosa cells	Bronchoscopy, biopsy of nasal cavity and small intestine	Retain the phenotypic and functional characteristics of the original tissue	Difficult to obtain in large quantities; Highly invasive biopsy procedure; Rapid aging/senescence in culture	Assessment of CFTR protein function; Personalized testing of targeted therapy	[16,17]
Immortalized cell lines	Lines derived from primary cells: from CF patient bronchi–CFBE41o-, 16HBE14o-, CFT1, etc.; wild-type–16HBE14o-, 9HTEo-, etc. Lines derived from tumor cells (e.g., Calu-3). Animal-derived cell lines (e.g., Fisher rat thyroid (FRT) cells)	Viral transformation, e.g., using SV40 Large T-antigen (SV40 LT)	Capable of unlimited division in culture; Enable genetic correction of the *CFTR* gene	Availability limited to a small number of common genotypes; Potential for genetic drift, simplified cellular architecture, and gradual loss of tissue-specific functions due to transformation and long-term cultivation	Drug screening; Study of synthesis, folding, degradation, and ion transport processes of mutant CFTR protein under controlled conditions; Preclinical efficacy assessment of therapies before advancing to animal models or scarce primary cells	[15,18,19]
Induced cellular cultures	iPSCs and their derivatives	Reprogramming of somatic cells (fibroblasts, blood cells) into a pluripotent state using Yamanaka factors: Oct4, Sox2, Klf4, c-Myc	Directed differentiation enables the generation of cells from difficult-to-access tissues without invasive procedures (e.g., lung epithelium); Unlimited cultivation allows for *CFTR* gene correction studies; Potential for CF cell therapy	Lengthy differentiation process (weeks to months); High cost; Epigenetic modifications from the original somatic cells may affect differentiation and function of derived cells	Creation of an unlimited source of patient cells with a unique genotype; Development of personalized therapies; Modeling CF pathogenesis by differentiating iPSCs into cells of affected organs (e.g., liver, pancreas, intestine) to study systemic manifestations of the disease; Correction of pathogenic variants using CRISPR/Cas9	[20,21]
Spheroids	Nasospheroids; Bronchospheroids; Alveolar spheroids	Culturing primary or induced cells in suspension	Simple to generate; Relatively rapid growth; More physiologically relevant than monolayer-cultured cells	Do not recapitulate epithelial polarity, differentiation, or structure; Lack a lumen, characteristic of organoids, complicating the study of ion and mucus transport; Absence of microenvironment and interactions with other cell types	Assessment of CFTR channel function, e.g., using the FIS assay; Potential for personalized drug testing	[22,23,24]
Organoids	Intestinal organoids; Respiratory tract organoids (nasal, bronchial, pulmonary, and alveolar)	Primary cells or iPSC-derived cells cultured in Matrigel	Share structural, functional, and compositional similarities—including cellular interactions, polarity, and microenvironment—with the target organ	Challenging cultivation and standardization; High cost and lengthy generation time	Study of CFTR function, inflammation, and cellular interactions; Personalized therapy selection	[21,25,26]
3D Bioprinting	Triple-layered alveolar barrier model; Lung airway model	Layer-by-layer fabrication of 3D constructs comprising cells and matrix using a bioprinter	Models macroscale tissue and organ-level processes; Recapitulates impaired mucociliary clearance	Not a primary tool for studying molecular mechanisms of CFTR channel function; High cost	Study of bacterial biofilm formation; Testing the efficacy of antimicrobials and CF therapies	[27,28,29]
Microfluidic technologies	Organ-on-a-chip; Organoid-on-a-chip	Fabrication of biochips with microchannel networks using techniques such as molding, soft lithography, and 3D printing	Enables comprehensive study of CF pathophysiology (inflammation, immune response, infection); Allow real-time monitoring via microsensors. Simulates mechanical forces (airflow, coughing); Suitable for high-throughput drug screening	Complexity in system manufacturing and operation; Organoid-on-a-chip technology remains under active development	Preclinical evaluation of therapy efficacy (e.g., CFTR correctors such as lumacaftor); Investigation of disease mechanisms in human tissue; Screening of anti-inflammatory and antimicrobial agents; Fundamental research, e.g., on fibrotic processes	[30,31,32,33]

List of abbreviations: iPSCs—induced pluripotent stem cells, CF—cystic fibrosis, FIS—Forskolin-Induced Swelling.

**Table 2:** Comparison of methods for assessing CFTR channel functional activity.

Method	Sample	Readout	Measurement type	Throughput	Sensitivity	Specificity	Applications	References
Patch-clamp	Individual cells	Direct measurement of ionic currents across the whole cell or through single CFTR channels	Direct	Very low	Very high	Very high	Detailed electrophysiological studies of single channels. Allows investigation of opening/closing kinetics, conductance, and the direct effects of drugs on CFTR. The “gold standard” for mechanistic research.	[60]
Ussing chamber	Cell layer (epithelium), cell monolayer, or organoids on transwells; ALI-cultured epithelial layers	Transepithelial ion current and potential difference	Direct	Low	High	High	Measures transepithelial ion transport across cell layers or tissue. The “gold standard” for functional validation of therapy efficacy (drugs, gene correction), often using protocols that block ENaC to isolate the CFTR-specific current.	[23,61]
Iodide-selective electrodes	Cell suspension	Rate of iodide efflux from cells	Indirect	Medium	Medium	Medium	Used for fundamental studies of ion transport kinetics and mechanisms	[62]
Halide-sensitive fluorescent probes	2D cell cultures, monolayers	Fluorescence changes due to ion concentration shift (CFTR-dependent iodide transport)	Indirect	High	Very high	High	Used for routine screening of CFTR modulators and for studying basic channel function in research	[63]
Halide-sensitive yellow fluorescent protein (HS-YFP) quenching	2D cell cultures, monolayers	Quenching of HS-YFP fluorescence upon iodide influx from cells	Indirect	High	High	Medium	High-throughput screening of CFTR potentiators/correctors in stable cell lines. A primary tool for pharmaceutical companies in the initial screening of large compound libraries.	[64,65]
Forskolin-Induced Swelling (FIS)	Organoids (intestinal, nasal, lung), spheroids	Organoid diameter as a result of ion and water influx	Indirect	High	Medium–high	High	Functional assay for patient-derived organoids. Used in personalized medicine—to predict response to CFTR modulators and assess the efficacy of gene editing.	[22,52,66]
Steady-state Lumen Area (SLA)	Organoids (intestinal and lung)	Lumen area (internal cavity) of the organoid	Indirect	High	Medium	Medium	Personalized medicine, preclinical drug screening, assessment of gene therapy efficacy	[67,68]

List of abbreviations: ALI—Air-Liquid Interface, HS-YFP—Halide-Sensitive Yellow Fluorescent Protein.

**Table 3:** Guidelines for selecting CFTR function assessment methods based on research objectives.

Research Objective		Primary Method	Validation Method	Key Selection Criteria	Cell Model	Time Requirement
Study of channel mechanism (channel biophysics, modulator action)		Patch-clamp (inside-out, whole-cell)	Iodide-selective electrodes	Maximal resolution (single-channel level), direct current measurement	Transfected cell lines, primary cells	Low: 1 day (automated patch-clamp), several days (manual)
Primary screening of correctors/potentiators (>10^3^ compounds)		HS-YFP assay, halide-sensitive fluorescent probes, automated patch-clamp	FIS assay in organoids (96-well plates)	Maximum throughput, automation, cost per compound	Immortalized cell lines → patient organoids	Low: (hours–days, automated)
Validation of candidates prior to clinical trials		Ussing chamber (ALI-cultures)	Ussing chamber (ex vivo biopsies)	Correlation with clinical endpoints (e.g., FEV_1_), physiological relevance	Primary bronchial/nasal epithelial cells	Medium, 3–4 weeks (ALI-culture) + 1 day (measurement)
Personalized therapy selection		FIS assay in intestinal organoids	FIS assay in nasal organoids (for monitoring)	In vitro–in vivo correlation, biomaterial availability, potential for repeated testing	Intestinal and respiratory organoids	Medium (days–weeks for culturing and analysis)
Assessment of gene therapy efficacy	Assessment of gene therapy with low correction efficiency (<15%)	Ussing chamber	FIS assay in organoids	Sensitivity to small fractions of corrected cells, tissue specificity	Edited primary cells or iPSC-derived cells, chimeric cultures	High (weeks–months for iPSC derivation and differentiation)
	Preclinical gene therapy screening	FIS assay in organoids (>10 genotypes)	Ussing chamber (validation of top candidates)	Screening of multiple genotypes, physiological relevance, throughput	Organoid biobank from patients with diverse mutations	Medium–High (days–weeks)

List of abbreviations: HS-YFP—Halide-Sensitive Yellow Fluorescent Protein, FIS—Forskolin-Induced Swelling, ALI—Air-Liquid Interface.

## Data Availability

Data sharing is not applicable to this article as no new data were created in this study.
